# Supplementary material for: Capturing ion trapping and detrapping dynamics in electrochromic thin films
Source: Nat Commun. 2024 Mar 14;15:2294. doi: 10.1038/s41467-024-46500-8 (PMC10937924; doi:10.1038/s41467-024-46500-8)
Supplement: Supplementary file 1 — Supplementary Information [file 41467_2024_46500_MOESM1_ESM.pdf]

Supplementary Information

Capturing ion trapping and detrapping dynamics in electrochromic thin films

Renfu Zhang[1], Qinqi Zhou[1], Siyuan Huang[1], Yiwen Zhang[1], Rui-Tao Wen[1, 2*]

[1]Department of Materials Science and Engineering, Southern University of Science and Technology, Shenzhen 518055, China

[2]Guangdong Provincial Key Laboratory of Functional Oxide Materials and Devices, Southern University of Science and Technology, Shenzhen, 518055, China

This file includes:

Supplementary Figures 1 to 24

Supplementary Tables 1 to 3

Supplementary Note 1

Supplementary References 1 to 9

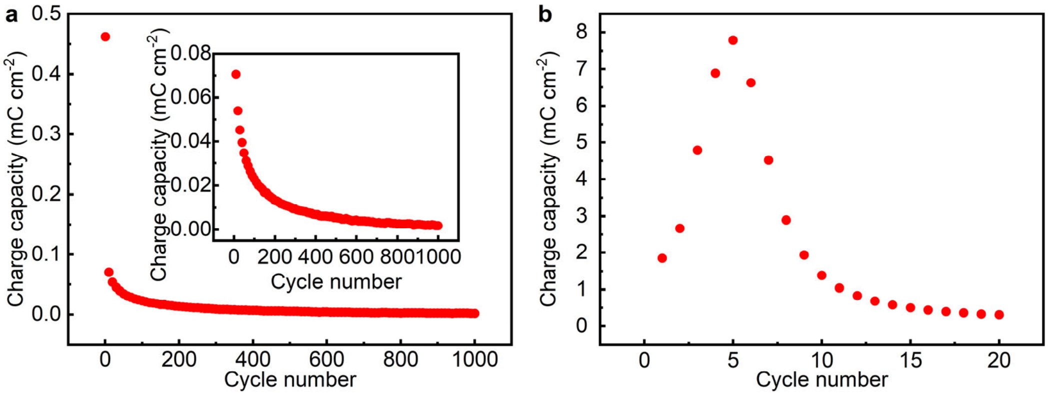

Supplementary Figure 1 $\boldsymbol{Q_{inserted}}$**-**$\boldsymbol{Q_{extracted}}$ **(*ΔQ*) versus cycle number, derived from CV data, indicating that ion trapping happens upon cycling. a**, Variation of **Δ*Q*** during CV cycling in the range 2.0-4.0 V with a sweep rate at 20 mV s^{-1}. Inset: data excludes the 1st cycle, which is especially high because of the formation of a SEI[1, 2, 3]; **b**, Variation of **Δ*Q*** during CV cycling in the range of 1.5-4.0 V with a sweep rate at 10 mV s^{-1}. It shows much higher charge capacity differences compared with **a**, suggesting a more rapid degradation.

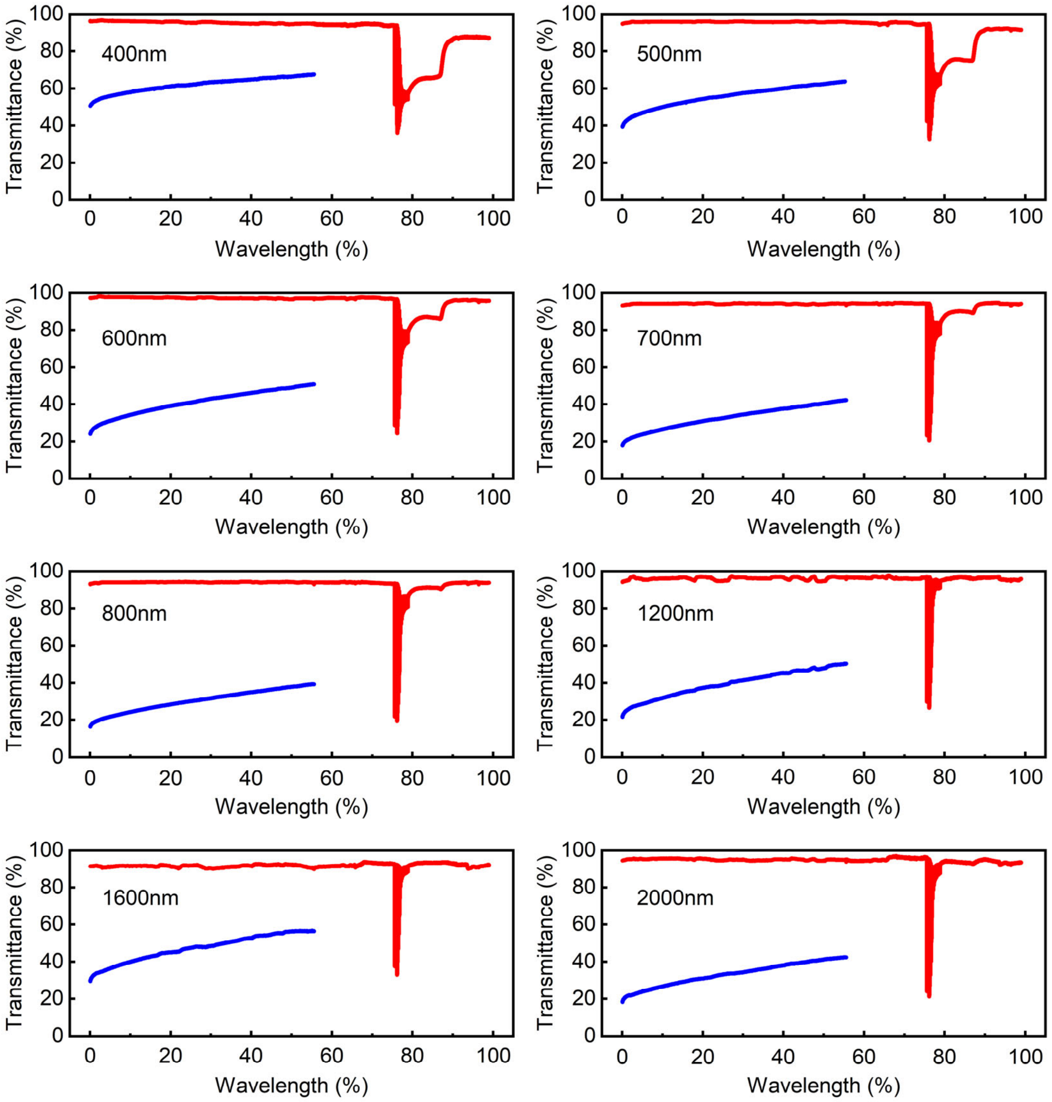

Supplementary Figure 2 *In-situ* optical transmittance at various wavelengths during the electrochemical processes as described in Fig. 1 of the main text. For profiles at 400 nm and 500 nm, the transmittances are partly un-rejuvenated because of the *irreversible* traps. For profiles at 1200 nm, 1600 nm and 2000 nm, the transmittances of the bleached state are hardly reduced even after severe trapping , this is because W atoms at this state exist mainly as W^{6+} and W^{4+}, and polaron hoping between W^{4+} and W^{6+} gives rise to optical absorption mainly in short-wavelength region. Proportions of each W state will be seen in Supplementary Table 1 and 2.

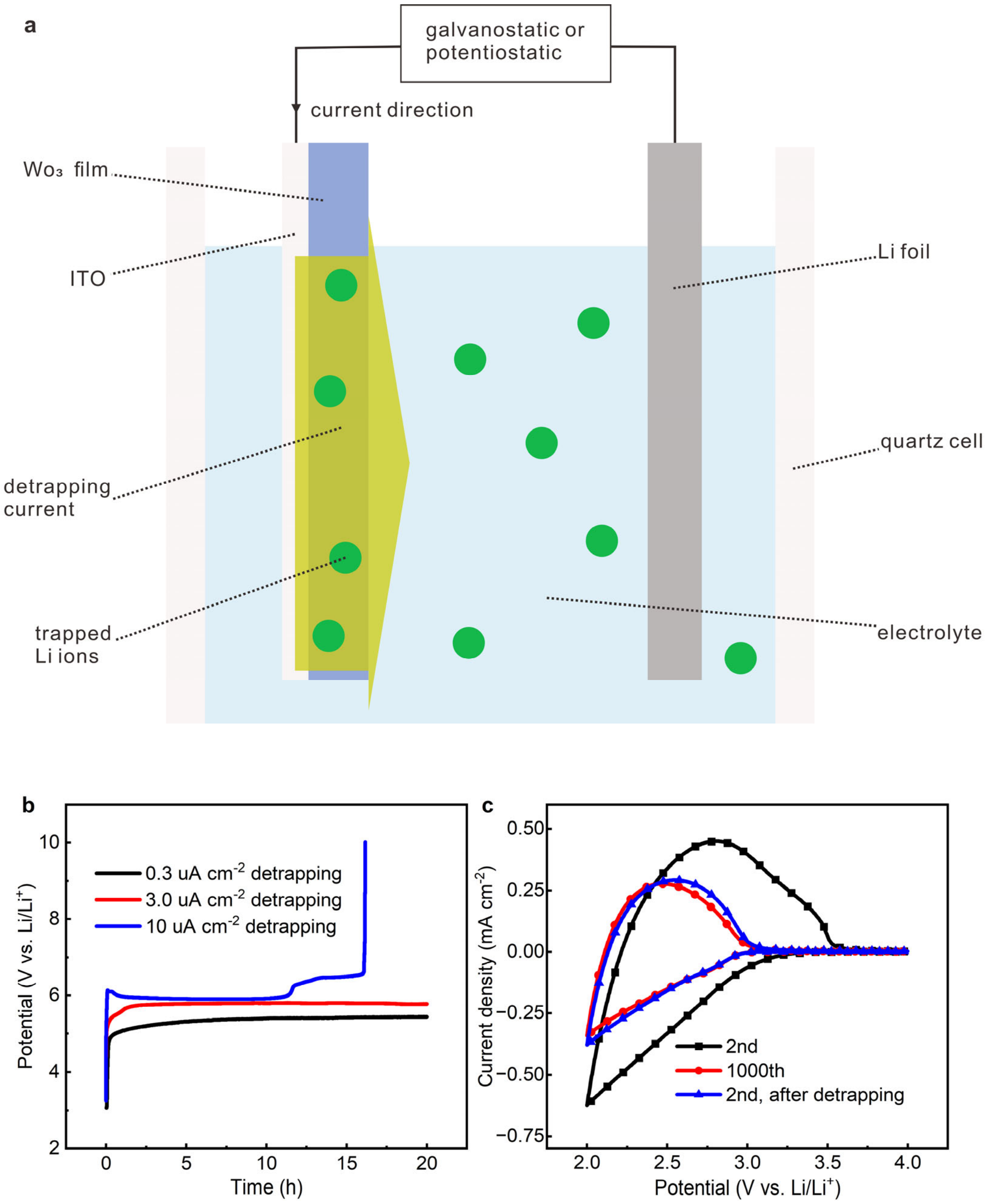

Supplementary Figure 3 Schematic diagram of detrapping procedure and electrochromic performance at different detrapping current densities. **a**, Schematic diagram of detrapping procedure, WO_3 films are coated on ITO/glass and are cycled either 1000 cycles at 2.0-4.0 V or 20 cycles at 1.5-4.0 V to yield severe degradation. Galvanostatic or potentiostatic detrapping is then imposed in the direction opposite to the one yielding Li ions insertion, which extracts the resident ions in the WO_3 electrodes. **b**, Potential profiles of different detrapping current densities, the loading of 10 μA cm^{-2} yields potential overflow while detrapping; on the other hand, 0.3 μA cm^{-2} is found to be inadequate because the film's performance scarcely recovers after detrapping, as shown in **c**, CV curves of the film before and after detrapping.

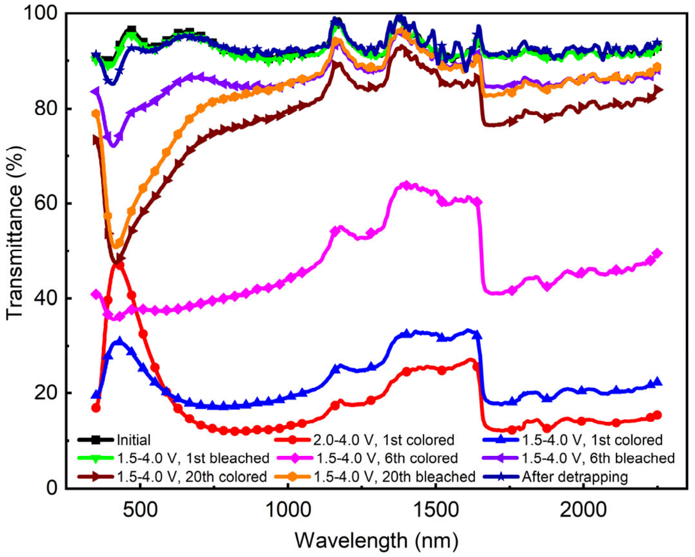

Supplementary Figure 4 Full spectra (350 nm-2250 nm) optical transmittance profiles showing transmittance valleys in the short-wavelength region (350 nm-500 nm). The 1st colored state at 2.0-4.0 V shows a broad peak in the short-wavelength region, and it is inhibited obviously at the 1st colored state at 1.5-4.0 V. This is because of the optical absorption originated from W^{4+} and W^{6+} polaron hoping resulted by the new formed W^{4+} at this state. On the other hand, the transmittance in the long-wavelength region of the 1st colored state at 1.5-4.0 V is higher than that of the 1st colored state at 2.0-4.0 V, despite the inserted charge of the former being much higher. This is because the reduction of W^{5+} amount as a cost of the formation of W^{4+}, leading to the reduction of polaron hoping between W^{5+} and W^{6+}, which accounts for the optical absorption in these wavelengths region. This tendency becomes more prevalent at the 6th colored and bleached states, and finally, at the 20th colored and bleached states, obvious valleys in the short-wavelength region can be seen, meanwhile, transmittances in the long-wavelength region are almost similar to that of the pristine state, because few amount of W^{5+} exists at these states. Proportions of each W state will be seen in Supplementary Table 1 and 2.

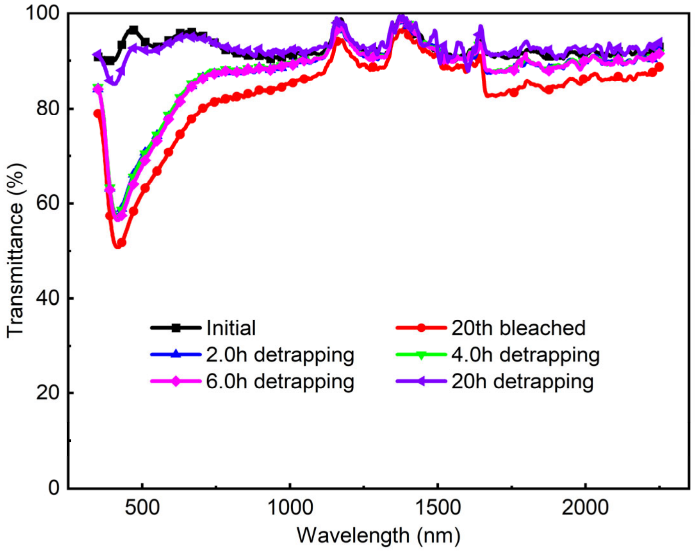

Supplementary Figure 5 Full spectra (350 nm-2250 nm) optical transmittance profiles for various detrapping processes after cycling in the range of 1.5-4.0 V with a scan rate of 10 mV s^{-1}. As shown from the 20th bleached state and after 2.0 h detrapping, the optical transmittance is slightly increasing immediately as the detrapping process is imposed, this is because of the release of independent W^{4+}. For states after 2.0 h detrapping, 4.0 h detrapping and 6.0 h detrapping, the optical transmittances are hardly altered. In other words, the optical transmittance reaches plateau. This plateau is assigned to the release of amorphous Li_2WO_4 (Supplementary Figure 22), since W atoms maintain the valency W^{6+}, so release of Li_2WO_4 does not change the efficiency of polaron hopping, and hence, the optical transmittance. Then the transmittance is rapidly increased to the initial state except for a small difference in the short-wavelength region, because of the release of W^{4+}-Li_2WO_4 coupling.

Supplementary Table 1 Proportions of each W valence at various states upon cycling in the range 2.0-4.0 V with a scan rate of 20 mV s^{-1}. The data are derived from XPS measurements. Only W^{6+} and W^{5+} (no W^{4+}) exist at these states. At the 5th colored state, abundant W^{6+} are reduced to W^{5+} because of the Li^{+} ion insertion, then polaron hoping between W^{6+} and W^{5+} drives the film colored. As the film is bleached, the proportions of W^{6+} and W^{5+} return to the pristine state because few ion accumulation at this stage. At the 1000th colored state, the proportion of W^{5+} is reduced as compared with the one at the 5th colored state because of ion trapping. Once the film is bleached, the proportions of W^{6+} and W^{5+} return to the pristine state, this indicates that the residing ions do not change the W valance and only suppress the formation of W^{5+} form W^{6+}. After detrapping, proportions of W^{6+} and W^{5+} are completely recovered.

State	W valance/%	
	W^{6+}	W^{5+}
**Initial**	89.989	10.011
**5th colored**	47.42	52.58
**5th bleached**	89.806	10.194
**1000th colored**	70.229	29.771
**1000th bleached**	89.559	10.441
**After detrapping**	89.846	10.154

Supplementary Note 1

It has been suggested[4] that, W^{4+} instead of W^{5+} sites are present in the as-deposited W oxide films in addition to the dominant amount of W^{6+} sites, due to the sub-stoichiometric characteristics of the films. Optical absorption occurs once W^{5+} sites are generated as a result of the reduction of the W^{6+} sites, when ions are inserted. Our observations here demonstrate that not any W^{4+} sites are detected in the as-deposited film.

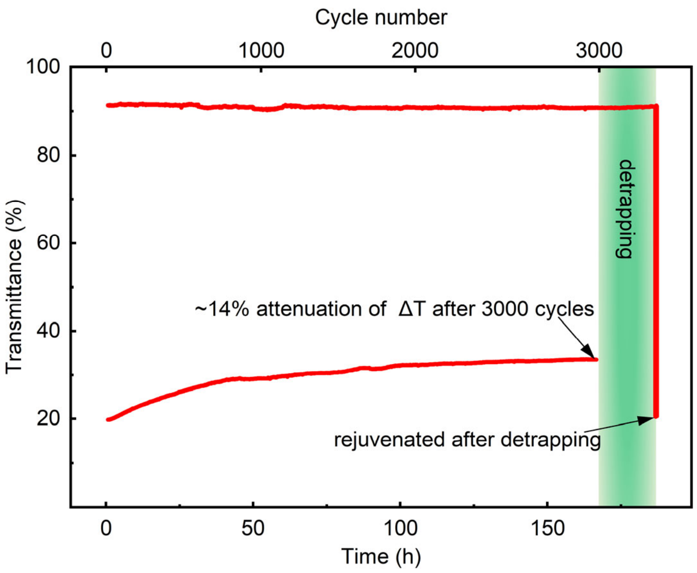

Supplementary Figure 6 The variation of ΔT of a dense WO_3 film during trapping/detrapping process. ~14% degradation is observed after 3000 cycles, which shows a greatly improved durability compared to the porous film used in the main text (~30% degradation after 1000 cycles). It can also be successfully rejuvenated, indicating that ion trapping/detrapping is general in WO_3.

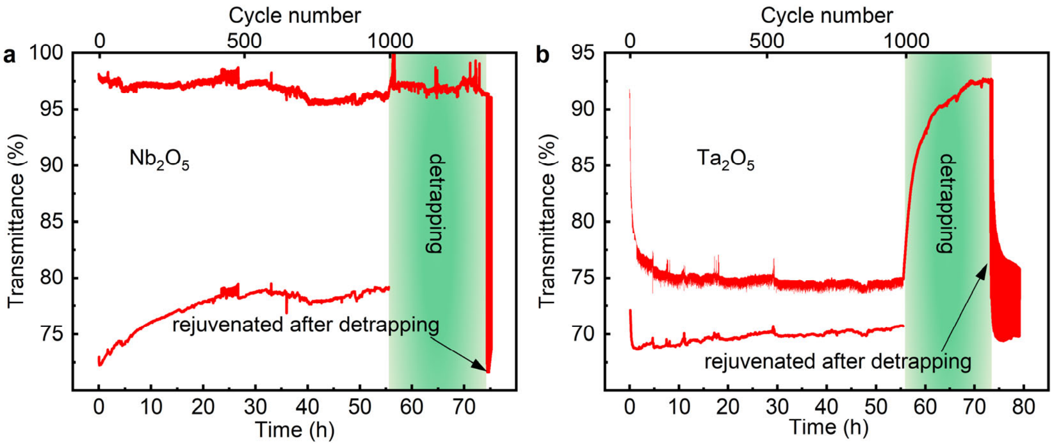

Supplementary Figure 7 Optical transmittances of Nb_2O_5 and Ta_2O_5 during ion trapping/detrapping processes. **a**, Nb_2O_5 is cycled in the range 1.7-3.7 V with 20 mV s^{-1} for 1000 cycles to yield clear degradation of colored state which shows a complete rejuvenation after galvanostatic detrapping for 20 hours; **b**, Ta_2O_5 is cycled in the range 1.3-4.0 V with 20 mV s^{-1} for 1000 cycles, with both the colored and bleached states degraded, which also shows a complete rejuvenation after galvanostatic detrapping for 20 hours. Together with WO_3 of this manuscript, TiO_2 and MoO_3 from the published papers, this indicates that trapping/detrapping process is generalizable and applies to all the cathodic electrochromic oxides.

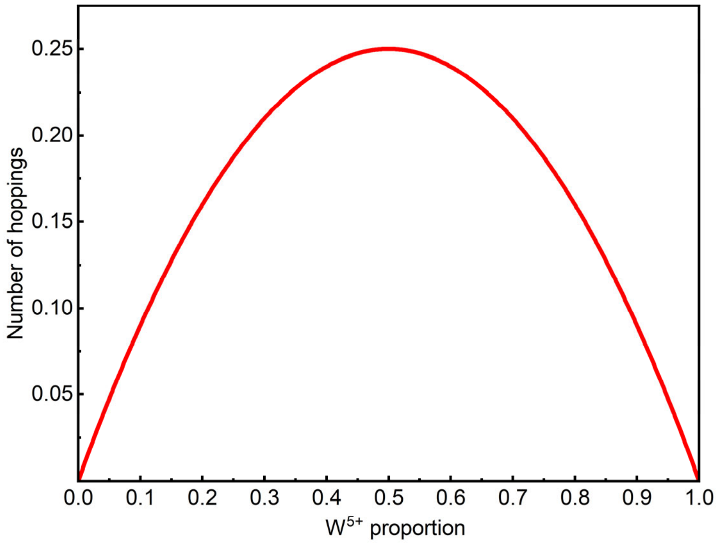

Supplementary Figure 8 Number of hopping transitions as a function of the W^{5+} proportion when only W^{5+} and W^{6+} are considered to be present in the WO_3 film. According to the site-saturation theory[5], if the probability of a site being W^{5+} is "*p*" at a certain stage of Li ion insertion, a site being W^{6+} is then (1-*p*) and the number of hopping transitions between W^{5+} and W^{6+} is *p*(1-*p*), this means the biggest number of hopping transitions will exist when the proportions of W^{5+} and W^{6+} are both 0.5.

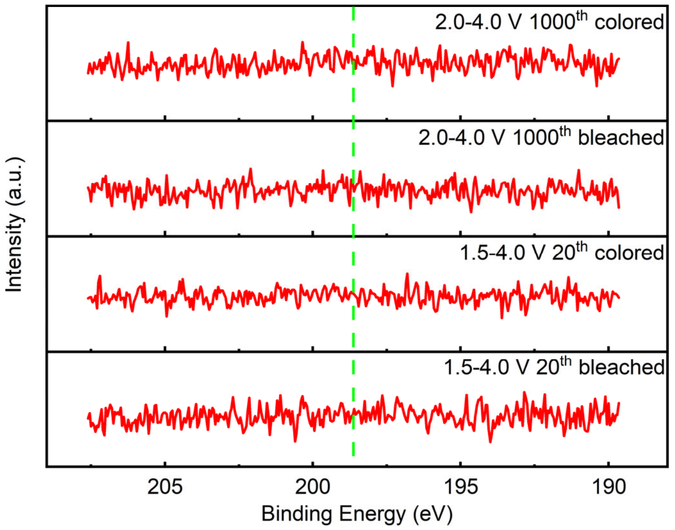

Supplementary Figure 9 XPS data of Cl 2p for films after severe trapping. Insets mark the corresponding states, *i.e.*, 1000th colored/bleached state after cycling in the range 2.0-4.0 V, and 20th colored/bleached state after cycling in the range 1.5-4.0 V. No signal from Cl 2p is detected, indicating that it is not involved in ion trappings.

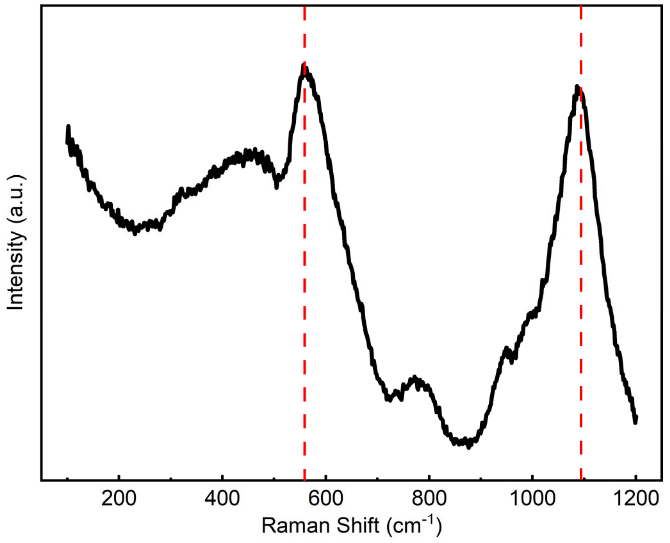

Supplementary Figure 10 Raman spectrum of bare ITO substrate. Peaks centered at ~560 cm^{-1} and ~1100 cm^{-1} in **Fig. 2** and **Fig. 3** of the main text are confirmed to originate from ITO.

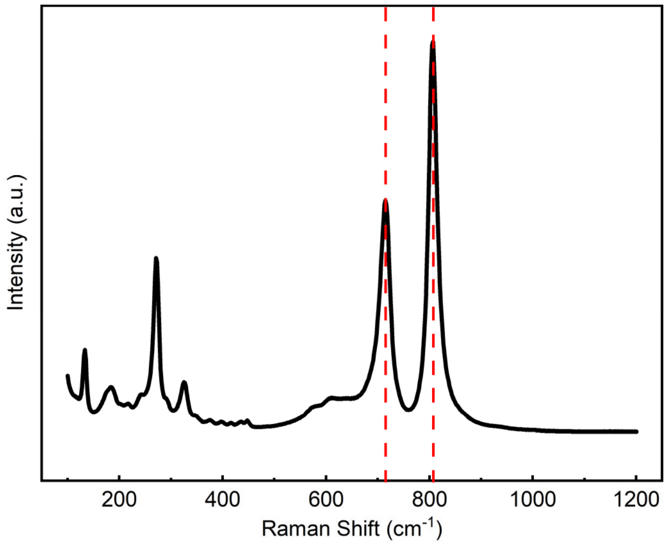

Supplementary Figure 11 Raman spectrum of commercial hexagonal nano WO_3 powders. Peaks centered at ~712 cm^{-1} and ~805 cm^{-1} are assigned to W^{6+}-O stretching modes of WO_3[6, 7], and the peak centered at ~950 cm^{-1} which is assigned to W^{6+}=O stretching modes[8, 9] is absent here due to the crystal characteristics of the sample.

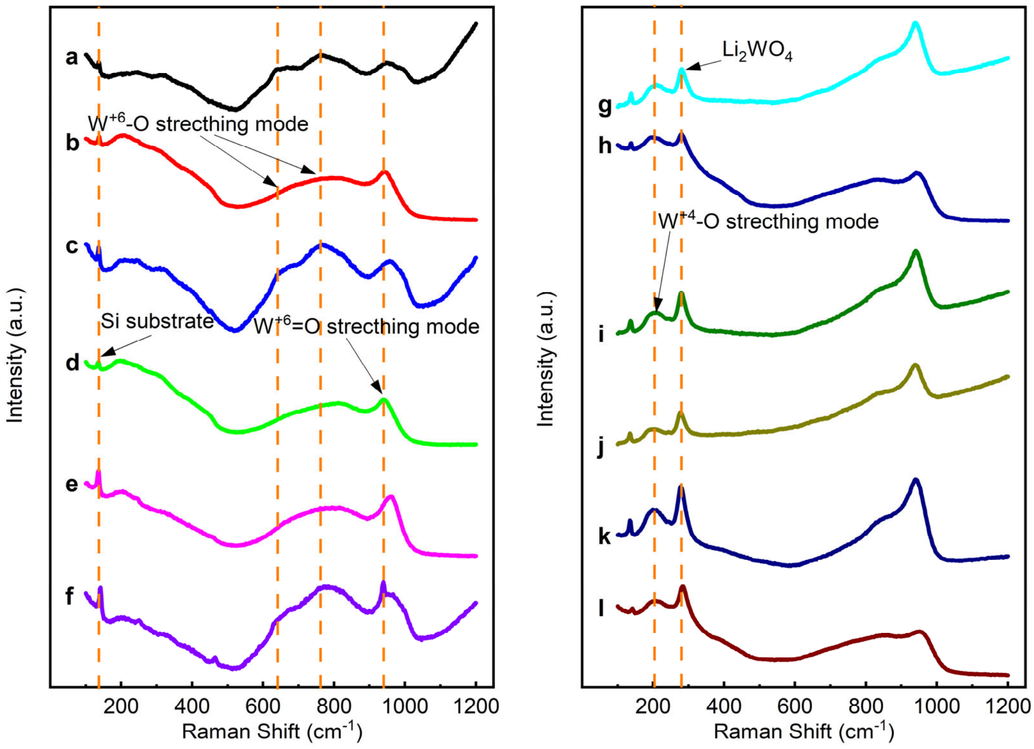

Supplementary Figure 12 Raman spectra of *a*-WO_3 deposited onto a W/Si substrate at various trapping/detrapping states, analogous to those shown in Fig. 3 of the main text. Results are same as those of WO_3 deposited onto ITO substrate, indicating that the ion trapping/detrapping species are related only to WO_3.

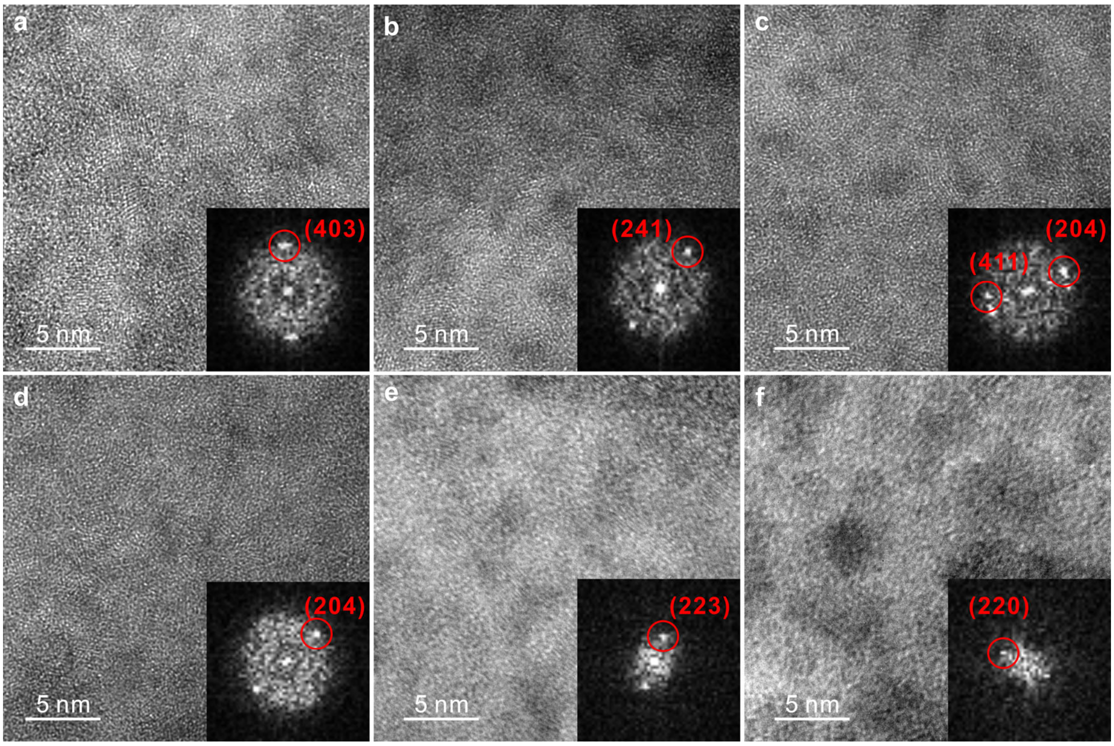

Supplementary Figure 13 HR-TEM images and the corresponding FFT patterns (insets) of various WO_3 samples at 1000th bleached state. In addition to **Fig. 2e** of the main text, more crystal planes are shown here, and the results are consistent that orthorhombic Li_2WO_4 were formed.

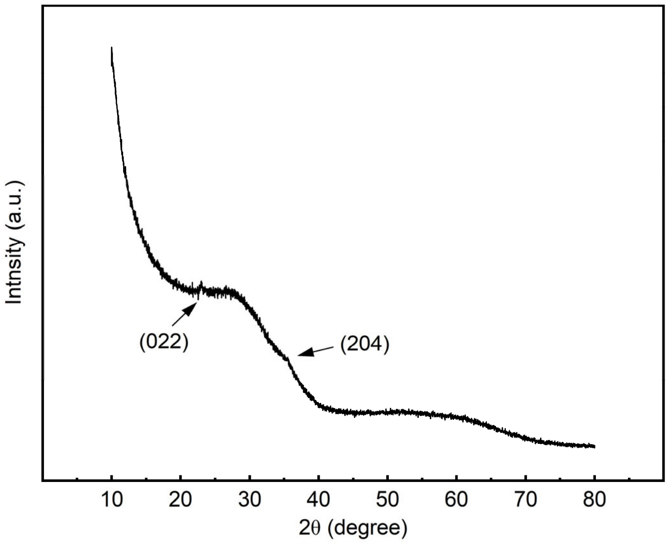

Supplementary Figure 14 XRD pattern based on grazing incidence of severely aged WO_3 film cycled within 2.0-4.0 V, 5 mV s^{-1}. Peaks emerging at 23° and 35.5° are assigned to (022) and (204) planes of orthorhombic Li_2WO_4, respectively, see JCPDS No: 28-0596. The results are consistent with the HR-TEM ones.

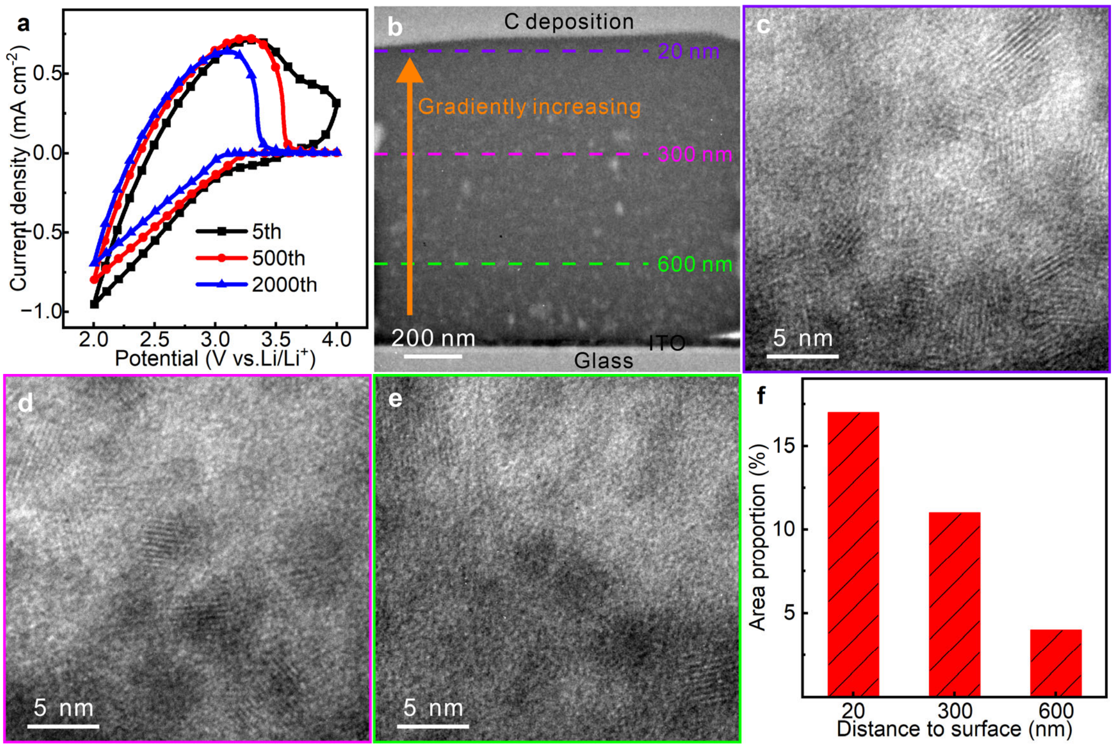

Supplementary Figure 15 HR-TEM images of a WO_3 thin film with a thickness of 800 nm at 2000th bleached state. It showed an incremental gradient distribution of Li_2WO_4 nanograins from low to the upper surface, as indicated by the arrow. **a**, CV curses of the 800 nm WO_3 film, showing the consistent degradation tendency as the 300 nm WO_3 films in this manuscript. **b**, Full image of the 800 nm WO_3 film by STEM, inset marks the position where HRTEM image is taken. **c-e**, HRTEM images of the position marked in **b**, showing an incremental gradient distribution of Li_2WO_4 nanograins. **f**, Gradient distribution of Li_2WO_4 nanograins derived from **c-e**, by segmenting the corresponding image into 10 x 10 lattices and counting the ones with nanograins.

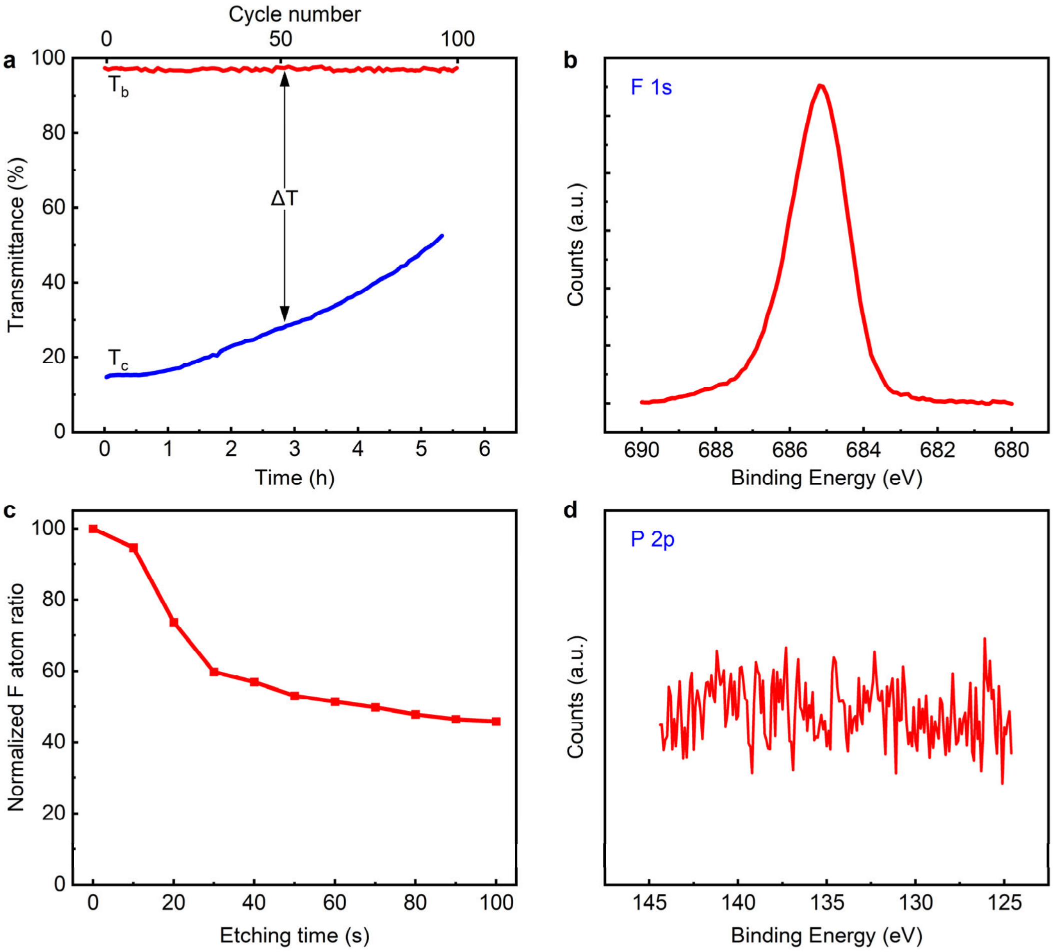

Supplementary Figure 16 *In-situ* optical transmittance and XPS investigations of WO_3 film cycled in 1.0 M $LiPF_6$-PC. **a**, *In-situ* optical transmittance at 550 nm of a WO_3 film cycled in 1.0 M $LiPF_6$-PC, showing the similar degradation of colored states and an accelerated degradation rate. **b**, XPS spectrum of F 1s and **c**, normalized F atom ratio as a function of etching time (distance to the surface) of WO_3 at 100th bleached state cycled in $LiPF_6$-PC, the gradient distribution near the surface indicates F is incorporated from the electrolyte. **d**, XPS data of P 2p of degraded film in $LiPF_6$-PC, no signal from P 2p is detected, indicating that it is not involved in ion trappings.

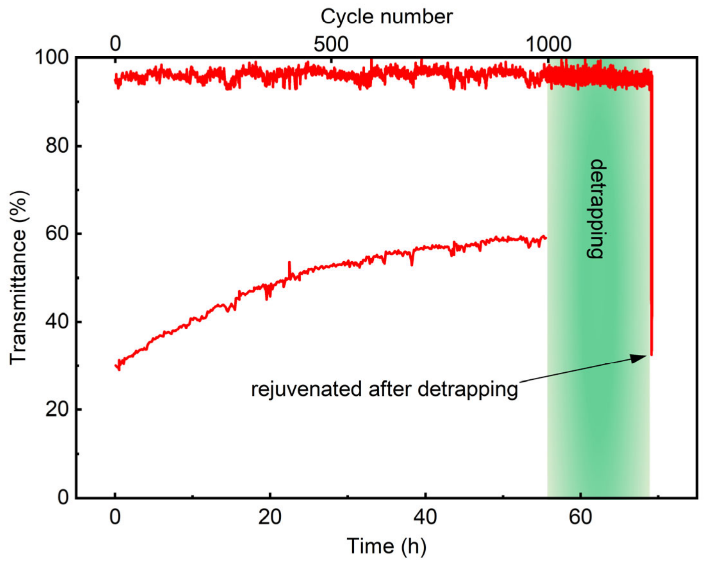

Supplementary Figure 17 *In-situ* optical transmittance of WO_3 film cycled in 1.0 M $LiClO_4$-ED/DEC. Similar results were yielded as compared to $LiClO_4$-PC, suggesting trapping/detrapping process is independent of solvent.

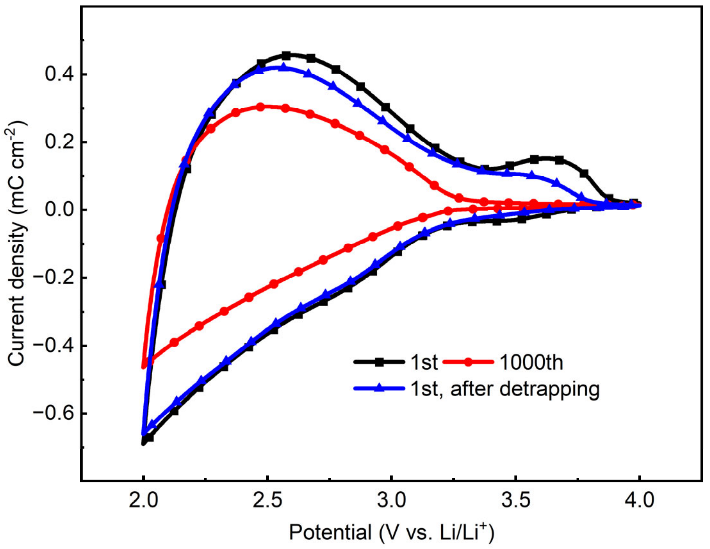

Supplementary Figure 18 CV curves for various states of WO_3 film on W/Si, showing the consistent trapping/detrapping process on an ITO/glass substrate. Because the substrate is Si/W, the optical transmittance is not recorded. Since ITO is replaced with Si/W where no extra oxygen can be provided, and the degradation is consistent with the one on ITO/glass, thus, ITO is excluded as the origin of extra oxygen.

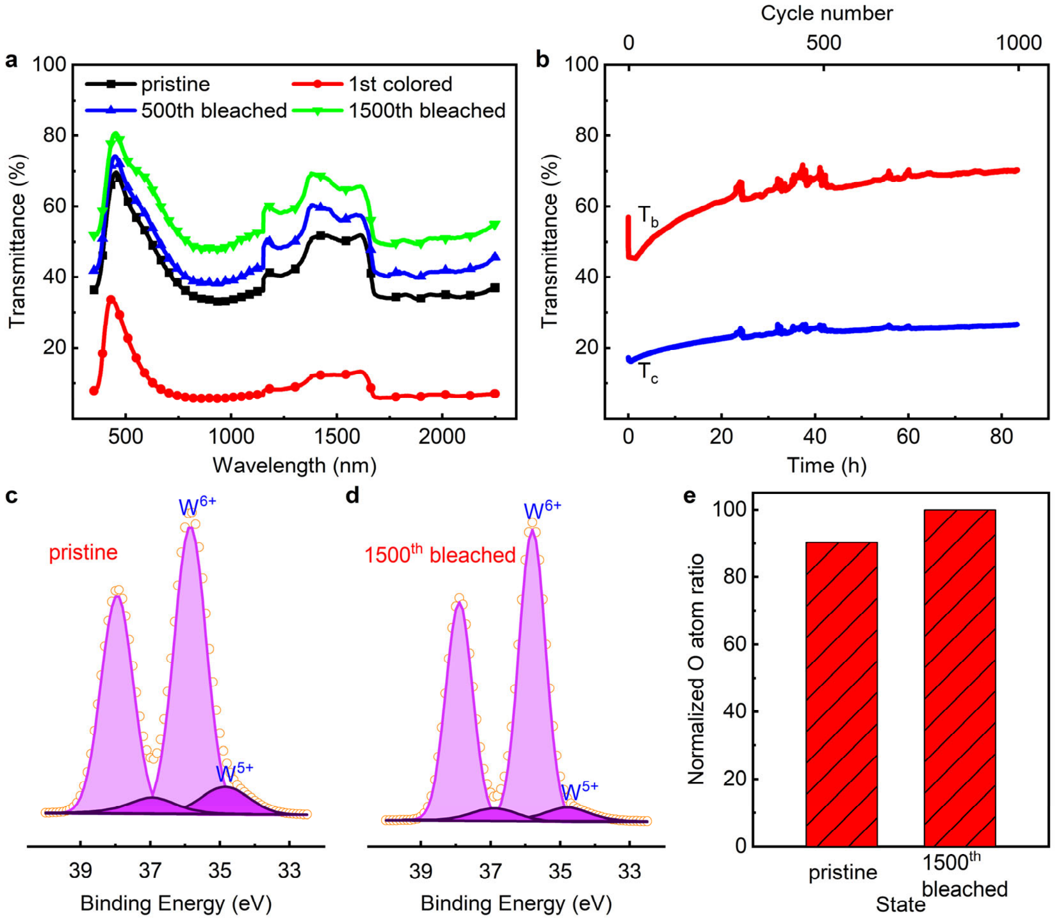

Supplementary Figure 19 ***In-situ*** **optical transmittance and XPS investigations on a WO_{3-z} film, indicating the oxygen is incorporated from electrolyte upon cycling.** **a**, Full transmittance profiles (350-2250 nm) at different states, and **b**, *In-situ* optical transmittance at 550 nm of the WO_{3-z} film, showing the increased transmittances at bleached states. **c**-**e**, XPS results of the WO_{3-z} film at pristine state, 1500th bleached state, and the normalized O atom ratio derived from these, respectively, showing that the bluish color at the pristine state is due to the existence of large number of oxygen vacancies induced W^{5+}, and the increased transmittance at bleached states upon cycling is due to oxidization from W^{5+} to W^{6+}.

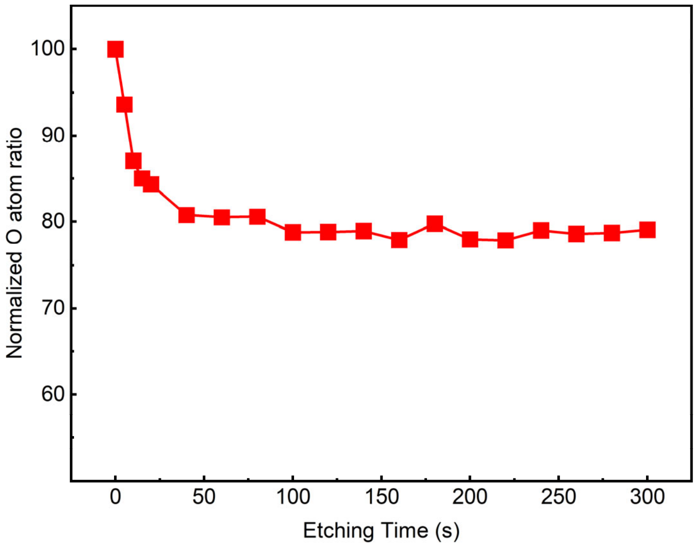

Supplementary Figure 20 Normalized O atom ratio of WO_3 film at 1000th bleached state, obtained with the assistance of XPS depth profile. It shows obvious decrease from film surface to ITO, indicating that oxygen is from the electrolyte, and is in good accordance with, also well explains, the TEM results which shows orthorhombic Li_2WO_4 nanograins concentrate in near the surface.

Supplementary Table 2 Proportions of W valence at various states upon cycling in the range 1.5-4.0 V with a scan rate of 10 mV s^{-1}. The data are derived from XPS measurements. W^{6+}, W^{5+} and W^{4+} sites exist at these states. At the 3rd colored state, part of the W^{4+} sites are formed at the cost of a decreasing W^{5+} concentration, as compared with the 5th colored state at 2.0-4.0 V, using a scan rate of 20 mV s^{-1}, this accounts for the flat of transmittance profile in short-wavelength region and the slight increase in long-wavelength region (Supplementary **Figure 4**). When bleached, the proportions of W^{6+} and W^{5+} return to the pristine state because of insignificant ion trapping at this stage. At the 6th bleached state, the proportion of W^{4+} further increases while the proportion of W^{5+} decreases due to the ion accumulation. At the 20th colored and bleached states, the formed W^{4+} are extremely accumulated and the proportions of W valence are hardly altered because of severe trapping at this stage. After detrapping, proportions of W^{6+} and W^{5+} almost return to the pristine state except for a few residual W^{4+} because of *irreversible* traps.

**State**	**W valance/%**		
	**W^{6+}**	**W^{5+}**	**W^{4+}**
**3th colored**	51.385	31.53	17.085
**3th bleached**	89.944	10.056	0
**6th bleached**	51.411	23.15	25.439
**20th colored**	37.417	13.214	49.369
**20th bleached**	41.079	15.555	43.366
**After detrapping**	85.09	8.878	6.033

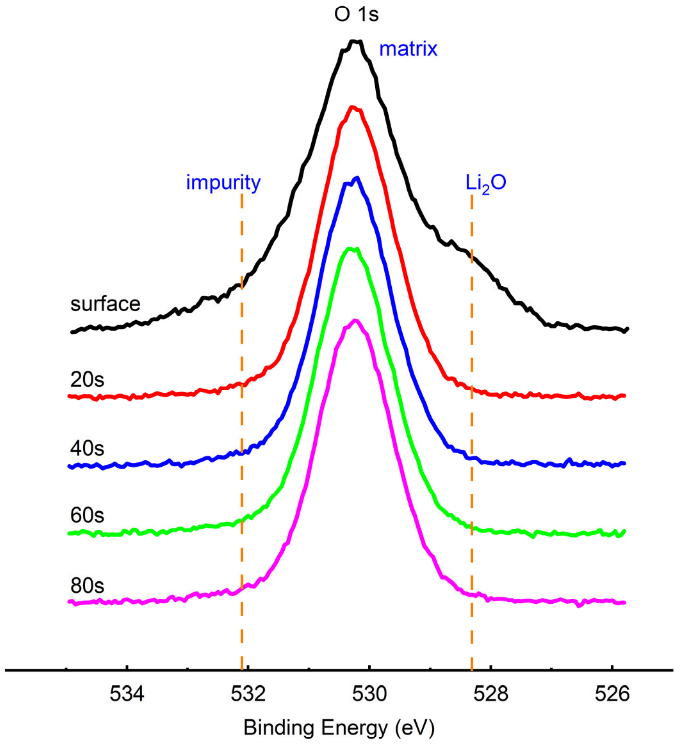

Supplementary Figure 21 XPS depth profile for 20th bleached state in 1.5-4.0 V. Peak centered at 528.3 eV is assigned to Li_2O, and is detected from the surface level only, indicating the Li_2O is limited to the very surface of the film.

Supplementary Table 3 Proportions of W valence at various states selected at marked points in Fig. 4 of the main text. The data are derived from XPS measurements. Point ***I*** is selected at the middle of the first milder step, the amount of W^{4+} at this state is obviously reduced compared with that of the 20th bleached (*i.e.*, severely trapped) state, revealing the release of independent W^{4+}. Points *II*, *III* and *IV* are selected to be just after the first mild step, at the middle of the plateau, and at the turning point before the second increase, respectively. Proportions of each W valence of these states hardly changes, this stage is assigned to release of amorphous Li_2WO_4. Point ***V*** is selected at the middle of second increase where the transmittance is largely recovered, the amount of W^{4+} is obviously reduced at this state, because of the decomposing the W^{4+}-Li_2WO_4 coupling.

State	W valance/%		
	W^{6+}	W^{5+}	W^{4+}
I	44.90979	26.03862	29.05159
II	52.06359	18.49683	29.43958
III	53.62209	17.30524	29.07267
IV	61.90216	12.35679	25.74106
V	79.2853	13.43988	7.27482

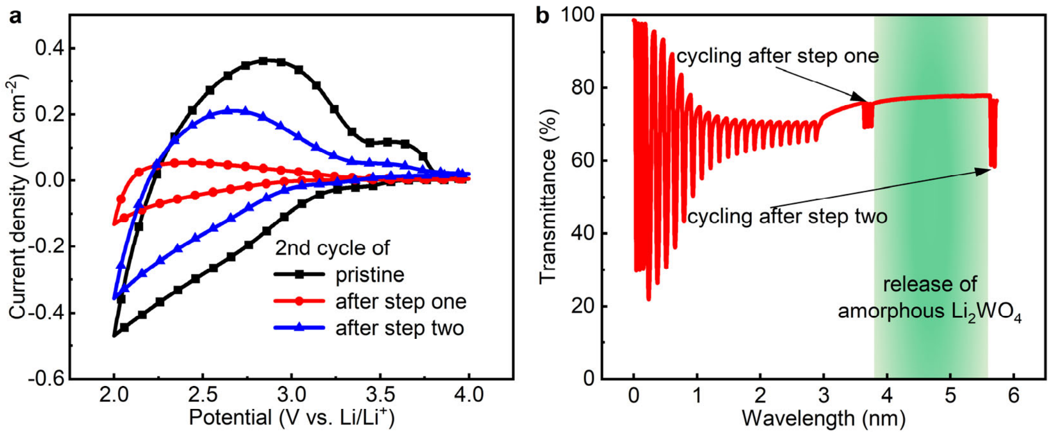

Supplementary Figure 22 Release of ions from amorphous Li_2WO_4 during the transmittance plateau period. **a**, CV curves of the 2nd cycle in the range 2.0-4.0 V with a sweep rate of 20 mV s^{-1} of the pristine state, after step one (***I***, as introduced in the main text, Performance degradation and rejuvenation), and after step two, respectively. An obvious recovery of the envelope area enclosed by the CV is noted after step two (***II***), the transmittance plateau period, revealing the release of ions from amorphous Li_2WO_4 during this period. **b**, *In-situ* transmittance recovery when cycled in the range of 2.0-4.0 V with a sweep rate of 20 mV s^{-1} after the plateau period, revealing again the release of ions from amorphous Li_2WO_4.

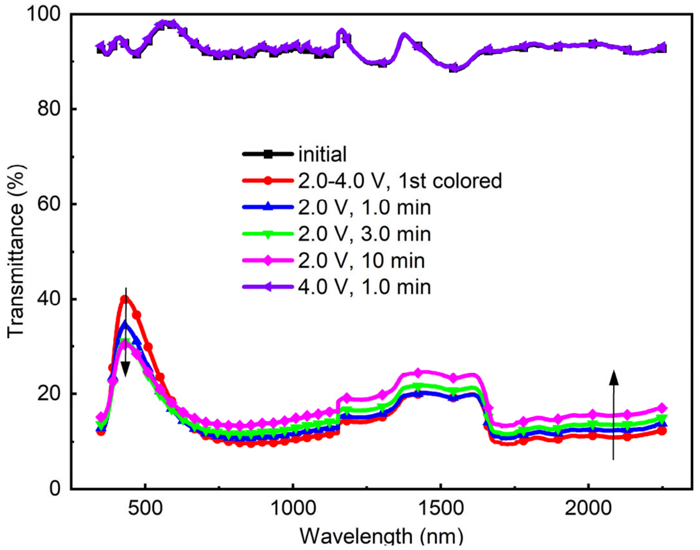

Supplementary Figure 23 *a*-WO_3 lithiated at 2.0 V for 1.0 minute, 3.0 minutes and 10 minutes, respectively, and imposed to a potentiostatic detrapping at 4.0 V. When the film is lithiated at 2.0 V, independent W^{4+} is formed, as proved by the decrease/increase of transmittance profiles in short-wavelength/long-wavelength, as well as the XPS and Raman results from the **Fig. 4c** and **4e**, 2.0 V, 1.0 min state of the main text. After potentiostatic detrapping at 4.0 V, the transmittance totally recovers to the pristine state, indicating that the independent W^{4+} can be totally released at 4.0 V.

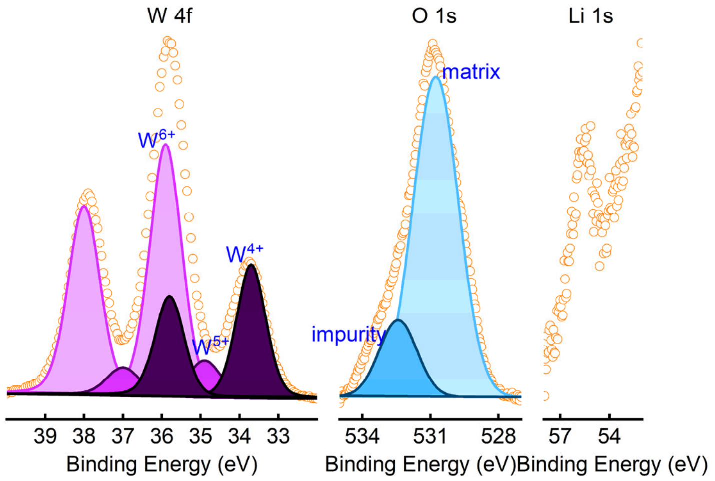

Supplementary Figure 24 *a*-WO_3 cycled in the range of 1.5-4.0 V at a scan rate of 10 mV s^{-1} for 20 cycles and imposed to a potentiostatic detrapping at 4.0 V. The W^{4+} and Li^+ signals reduced partly, while the Li_2O signal vanished, indicating that the coupled W^{4+}-Li_2O can be released at 4.0 V.

Supplementary References

1. Luo, D.; Zheng, L.; Zhang, Z.; Li, M.; Chen, Z. W.; Cui, R. G.; Shen, Y. B.; Li, G. R.; Feng, R. F.; Zhang, S. J.; Jiang, G. P.; Chen, L. W.; Yu, A. P.; Wang, X., Constructing

multifunctional solid electrolyte interface via in-situ polymerization for dendrite-free and low N/P ratio lithium metal batteries. *Nature Commun.* **2021**, *12*, 186.

2. Lin, R. Q.; He, Y. B.; Wang, C. Y.; Zou, P. C.; Hu, E. Y.; Yang, X. Q.; Xu, K.; Xin, H. L., Characterization of the structure and chemistry of the solid-electrolyte interface by cryo-EM leads to high-performance solid-state Li-metal batteries. *Nat. Nanotechnol.* **2022**, *17*, 768-776.

3. Chen, J.; Fan, X. L.; Li, Q.; Yang, H. B.; Khoshi, M. R.; Xu, Y. B.; Hwang, S.; Chen, L.; Ji, X.; Yang, C. Y.; He, H. X.; Wang, C. M.; Garfunkel, E.; Su, D.; Borodin, O.; Wang, C. S., Electrolyte design for LiF-rich solid-electrolyte interfaces to enable high-performance microsized alloy anodes for batteries. *Nat. Energy.* **2020**, *5*, 386-397.

4. Lee, S. H.; Cheong, H. M.; Tracy, C. E.; Mascarenhas, A.; Benson, D. K.; Deb, S. K., Raman spectroscopic studies of electrochromic a-WO_3. *Electrochimica Acta* **1999**, *44*, 3111-3115.

5. Berggren, L.; Jonsson, J. C.; Niklasson, G. A., Optical absorption in lithiated tungsten oxide thin films: Experiment and theory. *J. Appl. Phys.* **2007**, *102*, 083538.

6. Santato, C.; Odziemkowski, M.; Ulmann, M.; Augustynski, J., Crystallographically oriented mesoporous WO_3 films: synthesis, characterization, and applications. *J. Am. Chem. Soc.* **2001,** *123* (43), 10639-10649.

7. Pyper, O.; Kaschner, A.; Thomsenb, C., In situ Raman spectroscopy of the electrochemical reduction of WO_3 thin films in various electrolytes. *Solar Energy Materials and Solar Cells* **2002,** *71* (4), 511-522.

8. Bueno, P. R.; Pontes, F. M.; Leite, E. R.; Bulhões, L. O. S.; Pizani, P. S.; Lisboa-Filho, P. N.; Schreiner, W. H., Structural analysis of pure and $LiCF_3SO_3$-doped amorphous WO_3 electrochromic films and discussion on coloration kinetics. *J. Appl. Phys.* **2004,** *96* (4), 2102-2109.

9. Baserga, A.; Russo, V.; Fonzo, F. D.; Bailini, A.; Cattaneo, D.; Casari, C. S.; Bassi, A. L.; Bottani, C. E., Nanostructured tungsten oxide with controlled properties: Synthesis and Raman characterization. *Thin Solid Films* **2007,** *515* (16), 6465-6469.
