# Supplementary material for: Capturing ion trapping and detrapping dynamics in electrochromic thin films
Source: Nat Commun. 2024 Mar 14;15:2294. doi: 10.1038/s41467-024-46500-8 (PMC10937924; doi:10.1038/s41467-024-46500-8)
Supplement: Supplementary file 4 — Description of Additional Supplementary Files [file 41467_2024_46500_MOESM4_ESM.pdf]

DESCRIPTION OF ADDITIONAL SUPPLEMENTARY FILES

Supplementary Movie 1

Description: Screen recording to compare between colored states of 1.5-4.0 V at a scan rate of 10 mV s^{-1} and 2.0-4.0 V at a scan rate of 20 mV s^{-1}. CV at 2.0-4.0 V is carried out first and the transmittance profiles at the colored states are recorded. Then CV at 1.5-4.0 V is carried out next and the coloring process is shown in this movie. Transmittance in the short-wavelength region became flat while the transmittance in long-wavelength region increases, as discussed in Supplementary Figure. 4.
